# Late-Onset Radiation-Induced Leukoencephalopathy in a Long-Term Survivor with HER2-Positive Breast Cancer and Brain Metastases: A Case Report

**DOI:** 10.1159/cro/abzag022

**Published:** 2026-08-20

**Authors:** Miki Shimbara, Ritsuko Sasaki, Terufumi Kawamoto, Naoto Shikama, Misato Okazaki, Kotaro Ijima, Junichiro Watanabe, Goro Kutomi

**Affiliations:** a Department of Breast Oncology, Juntendo University Graduate School of Medicine, Tokyo, Japan; b Department of Breast Surgery, Institute of Science Tokyo, Tokyo, Japan; c Department of Radiation Oncology, Juntendo University Graduate School of Medicine, Tokyo, Japan

**Keywords:** Hippocampal-avoidance whole-brain radiotherapy, Brain metastases, HER2-positive breast cancer, Radiation-induced leukoencephalopathy, Case report

## Abstract

**Introduction:**

Recent advances in systemic therapies, particularly antibody-drug conjugates (ADCs), have substantially improved survival in patients with HER2-positive breast cancer and central nervous system (CNS) metastases. With increasing longevity, late adverse effects of cranial radiotherapy have emerged as clinically significant determinants of outcome.

**Case Presentation:**

A 43-year-old Japanese premenopausal woman with HER2-positive breast cancer developed multiple brain metastases 2 years after curative surgery and adjuvant chemotherapy and radiotherapy. She underwent hippocampal-avoidance whole-brain radiotherapy (HA-WBRT, 30 Gy in 10 fractions), followed by trastuzumab emtansine. Two years later, stereotactic radiotherapy (SRT, 30 Gy in 3 fractions) was administered for two new adjacent lesions. Six months later, leptomeningeal dissemination was detected, and trastuzumab deruxtecan was initiated, achieving a durable intracranial complete response. However, 4 years after HA-WBRT, she developed progressive cognitive decline and executive dysfunction, with the Mini-Mental State Examination score declining from a baseline of 30 to 18 within 2 months. Brain magnetic resonance imaging revealed diffuse, bilateral, relatively symmetric periventricular and deep white matter changes without evidence of tumor recurrence, consistent with late-onset radiation-induced leukoencephalopathy (RIL). Her neurological condition progressively deteriorated, with loss of social functioning and decision-making capacity, ultimately leading to discontinuation of active treatment and transition to best supportive care within 4 months of the diagnosis of RIL. She died approximately 2 months later, following progression of leukoencephalopathy.

**Conclusion:**

This case highlights that severe late-onset RIL can occur following HA-WBRT and salvage SRT in long-term survivors with breast cancer and CNS metastases. Although intracranial disease control was maintained for several years, progressive cognitive dysfunction led to discontinuation of active treatment. As survival improves in patients with breast cancer and CNS metastases, long-term management may need to consider not only intracranial disease control but also the risk of late neurological toxicity.

## Introduction

The prognosis of human epidermal growth factor receptor 2 (HER2)-positive breast cancer with central nervous system (CNS) metastases has historically been poor, with median survival reported to be approximately 11.5–18.9 months [1]. However, recent advances in systemic therapies, particularly antibody-drug conjugates (ADCs), have substantially improved intracranial disease control and overall survival [2]. As survival is prolonged, late adverse effects of cranial irradiation have emerged as clinically significant complications. Radiation-induced leukoencephalopathy (RIL) is a progressive and irreversible late toxicity characterized by demyelination and chronic neuroinflammation, leading to cognitive decline [3]. It typically develops months to years after whole-brain radiotherapy (WBRT), although very late presentations beyond 5–10 years have also been reported in long-term survivors [4]. Established risk factors include advanced age [5] and prior WBRT, particularly when WBRT is followed by stereotactic radiotherapy (SRT) [6, 7].

Here, we report a patient with HER2-positive recurrent breast cancer who achieved durable intracranial disease control after cranial irradiation and HER2-directed systemic therapy but developed late-onset RIL 4 years after hippocampal-avoidance WBRT (HA-WBRT). Neurological deterioration progressed over several weeks, resulting in profound cognitive decline with loss of self-awareness and ultimately precluding further cancer-directed therapy. This case highlights that delayed radiation-related neurotoxicity may become clinically apparent in long-term survivors despite sustained intracranial disease control.

## Case Presentation

A 43-year-old Japanese premenopausal woman with no significant past medical history and no family history of cancer noticed a mass in her right breast and was diagnosed with right breast cancer (cT2N0M0, stage IIA) [8]. She underwent a right mastectomy with axillary lymph node dissection. Histopathological examination showed invasive ductal carcinoma with nuclear grade 3 and histological grade III. Lymph node metastasis in 2/27 nodes was present. Immunohistochemical analysis revealed estrogen receptor-negative, progesterone receptor-negative, HER2 3+ and Ki-67 95%. Pathological stage was upstaged to pT3N1M0 (stage IIIA) [8]. Postoperatively, she received adjuvant chemotherapy consisting of four cycles of fluorouracil (600 mg/m^2^), epirubicin (100 mg/m^2^), and cyclophosphamide (500 mg/m^2^), followed by twelve weekly doses of paclitaxel (80 mg/m^2^) in combination with trastuzumab and pertuzumab for 1 year. Post-mastectomy radiation therapy was administered (50 Gy in 25 fractions) after chemotherapy. After completion of adjuvant therapy, she underwent regular follow-up every 6 months.

Two years after surgery, she presented with gait instability and headache. Brain magnetic resonance imaging (MRI) revealed seven brain metastases, including a 30-mm lesion in the cerebellar vermis causing mild brainstem compression and narrowing of the fourth ventricle, as well as several smaller lesions in the temporal and occipital lobes (Fig. 1a, b). Positron emission tomography-computed tomography (PET-CT) revealed no evidence of extracranial metastasis. Given the number and distribution of metastases and concerns about excessive brainstem irradiation with SRT, HA-WBRT was selected to preserve cognitive function and was delivered at a total dose of 30 Gy in 10 fractions (Fig. 1c). Mini-Mental State Examination (MMSE) score was 30 before HA-WBRT. Memantine was not administered prophylactically at the time of HA-WBRT because its use for this indication was not approved in Japan. Trastuzumab emtansine (T-DM1) (3.6 mg/kg) was initiated 2 weeks after completion of HA-WBRT. Two years after HA-WBRT, follow-up MRI revealed two new metastatic lesions in the left frontal lobe, measuring 5 mm and 3 mm, respectively (Fig. 1d). T-DM1 was temporarily withheld, and SRT was delivered a week later at a total dose of 30 Gy in 3 fractions (Fig. 1e). T-DM1 was resumed 4 weeks after completion of SRT. Six months later, leptomeningeal dissemination was detected (Fig. 1f). Systemic therapy was switched to trastuzumab deruxtecan (T-DXd). Due to grade 2 thrombocytopenia, the dose was reduced to 3.2 mg/kg, and she achieved and maintained a complete intracranial response for approximately 1.5 years.

**Fig. 1.**
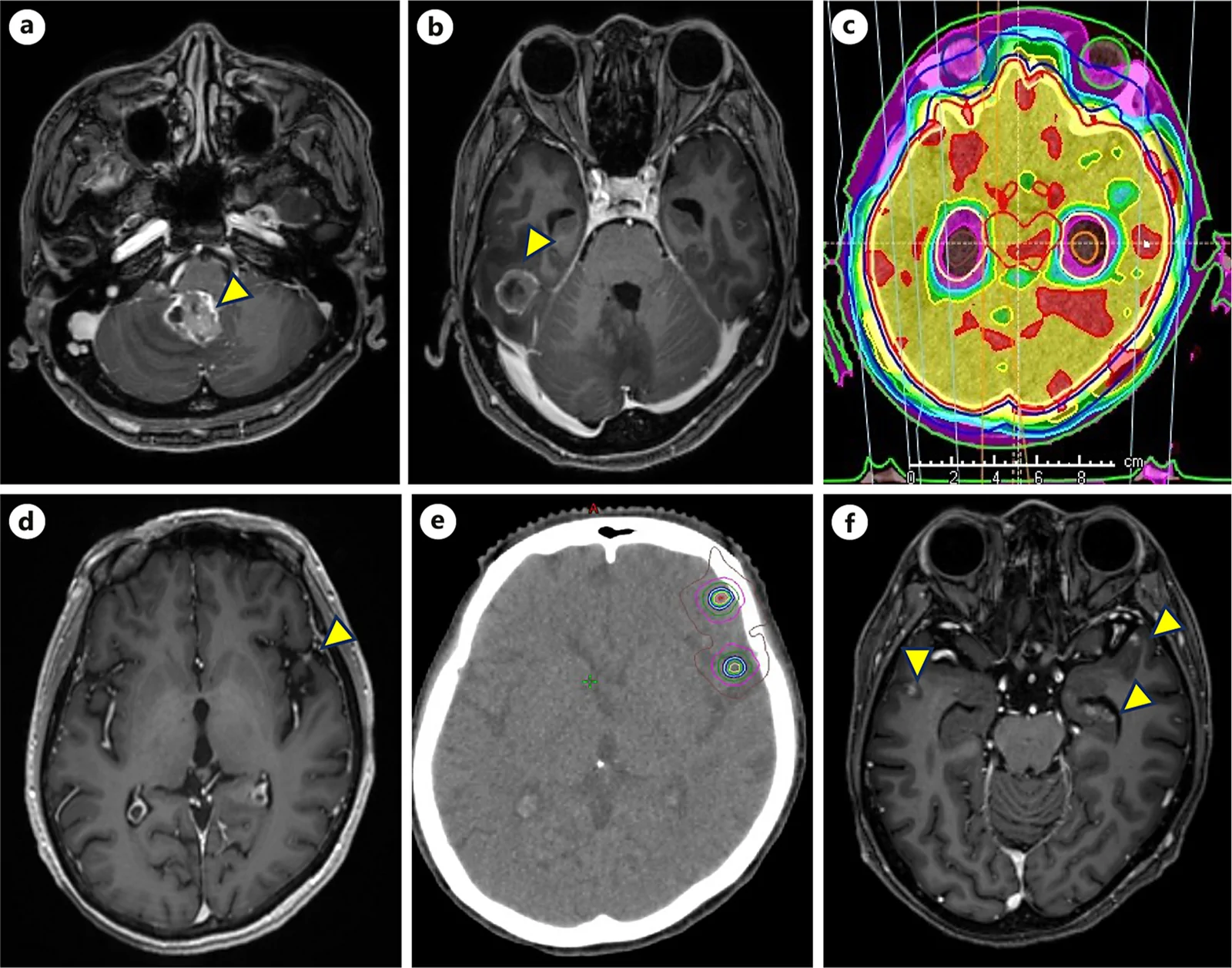
Serial neuroimaging findings during the clinical course. **a**, **b** Contrast-enhanced MRI obtained 2 years after surgery showed multiple brain metastases. **a** A 30-mm lesion was present in the cerebellar vermis, causing mild compression of the brainstem and narrowing of the fourth ventricle. **b** A 20-mm enhancing lesion was identified in the right temporal lobe. **c** Planning CT images for HA-WBRT administered at 30 Gy in 10 fractions. **d** Two years after HA-WBRT, a new 5-mm metastatic lesion developed in the left frontal lobe and was treated with SRT. **e** Planning CT images for SRT to two adjacent left frontal lobe lesions measuring 5 mm and 3 mm, delivered at 30 Gy in 3 fractions. **f** Contrast-enhanced MRI obtained 2.5 years after HA-WBRT demonstrated leptomeningeal dissemination. Arrowheads indicate metastatic lesions. MRI, magnetic resonance imaging; CT, computed tomography; HA-WBRT, hippocampal-avoidance whole-brain radiotherapy; SRT, stereotactic radiotherapy; Gy, gray.

Four years after HA-WBRT and 2 years after SRT, she developed progressive memory impairment, resulting in a decline in her ability to manage medications and perform occupational tasks. Neurocognitive assessment at symptom onset showed impaired performance (Fig. 2): MMSE score 25, compared with a consistently normal baseline of 30 on serial assessments performed every 3–4 months, Montreal Cognitive Assessment (MoCA) 22, and Frontal Assessment Battery (FAB) 10. Her cognitive function worsened markedly over the following months; the MMSE and MoCA further declined to 18 and 16, respectively. Less than 4 months after symptom onset, neurocognitive assessments were no longer feasible because of impaired responsiveness. She subsequently developed gait disturbance and loss of independence in activities of daily living.

**Fig. 2.**
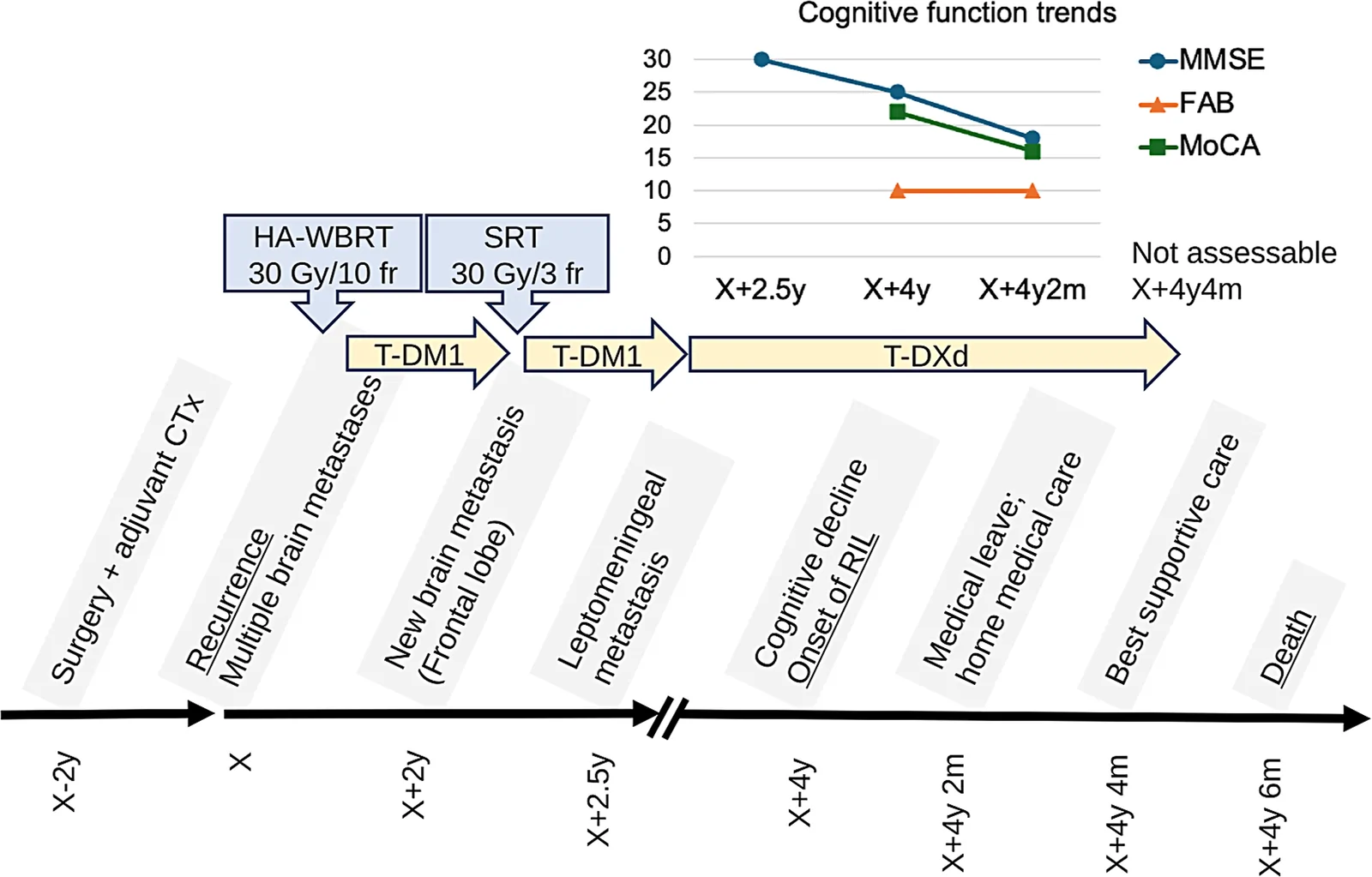
Clinical timeline and longitudinal cognitive function trends. The timeline summarizes the clinical course from initial surgery, with recurrence denoted as X years, through the development of brain and leptomeningeal metastases to the terminal phase. HA-WBRT and SRT were administered for intracranial disease during systemic therapy. After the onset of RIL at X + 4 years, cognitive function declined markedly, as reflected by serial decreases in MMSE, FAB, and MoCA scores, and eventually became unassessable. The patient was transitioned to best supportive care and died 6 months after the onset of RIL (X + 4 years 6 months). CTx, chemotherapy; HA-WBRT, hippocampal-avoidance whole-brain radiotherapy; SRT, stereotactic radiotherapy; T-DM1, trastuzumab emtansine; T-DXd, trastuzumab deruxtecan; MMSE, Mini-Mental State Examination; FAB, Frontal Assessment Battery; MoCA, Montreal Cognitive Assessment; RIL, radiation-induced leukoencephalopathy; fr, fraction (s).

Contrast-enhanced brain MRI showed no evidence of tumor recurrence or new metastatic lesions. However, T2-weighted images demonstrated diffuse, bilateral, relatively symmetric periventricular and deep white matter hyperintensities and generalized cerebral atrophy (Fig. 3a–f). Given the delayed onset after cranial irradiation, clinical course, and characteristic imaging pattern in the absence of tumor progression, the findings were considered most consistent with late-onset RIL. Although drug-associated leukoencephalopathy, including possible ADC-associated toxicity, was considered in the differential diagnosis, the imaging features were predominantly consistent with RIL [5]. Neurological evaluation, including laboratory assessment for infectious, metabolic, autoimmune, or paraneoplastic disorders, did not identify an alternative cause of the progressive neurological deterioration.

**Fig. 3.**
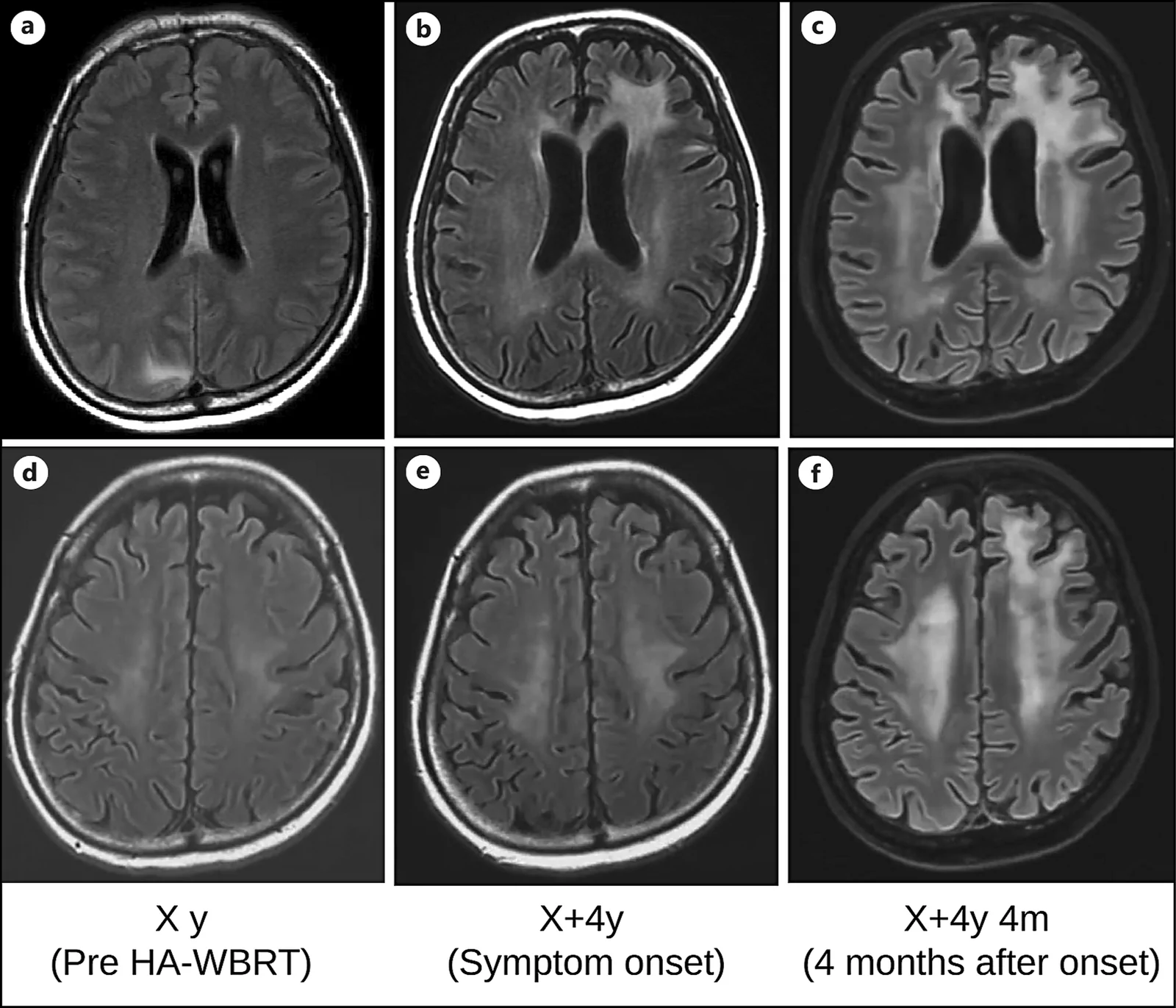
Serial brain MRI findings showing the progression of RIL. Axial FLAIR images at the level of the lateral ventricles (**a–c**) and centrum semiovale (**d–f**) demonstrate progressive diffuse periventricular white matter hyperintensity and generalized cerebral atrophy without evidence of tumor recurrence, consistent with RIL. **a**, **d** Pre-HA-WBRT. **b**, **e** At symptom onset. **c**, **f** Four months after symptom onset. MRI, magnetic resonance imaging; FLAIR, fluid-attenuated inversion recovery; RIL, radiation-induced leukoencephalopathy; HA-WBRT, hippocampal-avoidance whole-brain radiotherapy.

Following consultation with neurology, amantadine was initiated as a symptomatic treatment. However, her neurological status continued to decline, leading to discontinuation of systemic anticancer therapy. Within 4 months after the diagnosis of RIL, she lost decision-making capacity, and subsequent management decisions were made in consultation with her family. She was transitioned to best supportive care, subsequently became bedridden, and was unable to maintain oral intake. She died approximately 2 months after the transition to best supportive care. The overall clinical course was considered consistent with Common Terminology Criteria for Adverse Events grade 5 leukoencephalopathy.

## Discussion

This case highlights late-onset RIL as an increasingly relevant clinical challenge in the era of prolonged survival for metastatic breast cancer. Recent advances in systemic therapy have markedly improved outcomes for patients with HER2-positive metastatic breast cancer [9], including those with CNS metastases. Against this backdrop, T-DXd has demonstrated substantial and durable intracranial activity; in the ROSET-BM study, the median progression-free survival was 14.6 months, and the 24-month overall survival rate was 56.0% in patients with brain metastases [2, 10]. These improvements in systemic therapy and intracranial disease control may also increase the number of patients who survive long enough for delayed radiation-related neurotoxicity to become clinically apparent.

Late-delayed complications after cranial irradiation include RIL and radiation necrosis, which are distinct entities with different clinicoradiological features. RIL reflects diffuse white matter injury involving chronic neuroinflammation and glial injury and typically presents with progressive cognitive and functional decline in a frontal-subcortical pattern; management is largely supportive because no established disease-modifying therapy exists [3]. In the present case, contrast-enhanced MRI showed no evidence of tumor recurrence, whereas FLAIR/T2-weighted imaging demonstrated diffuse periventricular white matter hyperintensity with generalized cerebral atrophy. Notably, the characteristic sparing of subcortical U-fibers was consistent with a diagnosis of RIL (Fig. 3c) [11, 12]. Cognitive and functional symptoms worsened over a period of weeks in this case; however, the rate of clinical progression after RIL onset has rarely been quantitatively evaluated in previous studies [7, 13, 14]. By contrast, radiation necrosis is generally a more focal process driven predominantly by vascular injury and microcirculatory impairment, often presenting with localized neurological deficits or seizures and sometimes requiring advanced imaging or histopathology for confirmation [15]. Corticosteroids are commonly used, and bevacizumab or surgical intervention may be considered in selected cases [16].

Regarding risk factors for RIL, both patient- and treatment-related factors have been reported. Patient-related factors include advanced age and vascular comorbidities [5]. Among treatment-related factors, the combination of WBRT and subsequent SRT has been associated with a higher incidence and greater severity of leukoencephalopathy than SRT alone; the 3-year cumulative incidence of grade ≥2 leukoencephalopathy has been reported as 23.4% with WBRT plus SRT versus 5.7% with SRT alone [7]. In addition, a prior report identified a history of WBRT and the presence of multiple target lesions during SRT as independent risk factors, whereas cumulative radiation dose was not an independent predictor [7]. This patient had undergone HA-WBRT followed by salvage SRT to multiple lesions and therefore had recognized risk factors for the development of RIL. Moreover, these findings suggest that RIL pathogenesis involves complex mechanisms beyond simple dose accumulation and that salvage strategies following HA-WBRT remain inadequately standardized regarding dose fractionation and treatment sequencing. In the current era of effective systemic therapies, the timing and sequencing of cranial irradiation may therefore warrant more individualized optimization [17].

To mitigate neurocognitive toxicity, contemporary guidelines recommend HA-WBRT combined with memantine. The phase III NRG CC001 trial demonstrated better preservation of cognitive function with this approach compared with conventional WBRT [18]. However, even the final analysis of the trial had a median follow-up of only 12.1 months among surviving patients, leaving very late neurotoxicity in long-term survivors insufficiently characterized [19]. Our case highlights that fatal diffuse leukoencephalopathy can still occur several years after HA-WBRT. In Japan, memantine is approved only for Alzheimer’s disease, and its off-label use for radiation-related cognitive decline is not covered by national health insurance. Consequently, in our case, amantadine was initiated after a neurological consultation to address frontal-subcortical dysfunction associated with RIL, although supporting evidence for this approach remains limited. Recent structural imaging data suggest that HA-WBRT provides focal hippocampal protection but does not fully prevent global or extra-hippocampal structural changes. In addition, a recent randomized trial reported better symptom burden and functional outcomes with SRT than with HA-WBRT in selected patients with 5–20 brain metastases [20, 21]. Drug-induced leukoencephalopathy was also considered in the differential diagnosis. However, the clinical course was less typical of a primary drug-related neurotoxicity. While chemotherapy-associated leukoencephalopathy frequently presents acutely or subacutely after exposure and is often reversible, this case showed a delayed onset with progressive and irreversible deterioration [22, 23]. Conventional agents associated with leukoencephalopathy, such as cisplatin, cytarabine, and methotrexate, were not used in this case [24]. Although taxane-based chemotherapy and 5-fluorouracil were administered in the perioperative setting, the interval of more than 4 years before symptom onset argues against a causal relationship [25]. Among the administered ADCs, both T-DM1 and T-DXd were considered in the differential diagnosis, particularly T-DXd, which had been administered for approximately 1.5 years before symptom onset. However, pharmacovigilance data have not established leukoencephalopathy as a recognized adverse effect of either agent [26], and our literature search identified no clinical reports linking either agent to leukoencephalopathy or to the development or progression of RIL. Therefore, their contribution to the neurological deterioration in the present case could not be determined.

This case report has several limitations. First, a definitive pathological diagnosis could not be established because an autopsy was not performed. Second, cerebrospinal fluid analysis and advanced neuroimaging, such as amino acid PET, perfusion imaging, or MR spectroscopy, were not performed, limiting diagnostic confidence, particularly given the patient’s prior history of leptomeningeal dissemination.

## Conclusion

This case highlights that severe late-onset RIL can occur following HA-WBRT and salvage SRT in a long-term survivor with breast cancer and CNS metastases. Although intracranial disease control was maintained for several years, progressive cognitive dysfunction led to discontinuation of active treatment. Long-term management may need to consider not only durable intracranial disease control but also the risk of delayed neurological toxicity.

## Acknowledgment

We appreciate Katrin Ishii-Schrade (Juntendo University) for English language editing.

## Statement of Ethics

Written informed consent for publication of this case report and the accompanying images was obtained from the patient’s sister, as the patient had already passed away. Ethical approval is not required for this study in accordance with local or national guidelines. The CARE Checklist has been completed by the authors for this case report and is provided as online supplementary material (for all online suppl. material, see https://doi.org/10.1159/cro/abzag022).

## Conflict of Interest Statement

The authors declare no conflicts of interest.

## Funding Sources

This study was not supported by any sponsor or funder.

## Author Contributions

M.S. and R.S. contributed to conceptualization and writing of the original draft. T.K. and N.S. contributed to radiotherapy treatment decision-making and patient management and reviewed the manuscript. M.O., K.I., J.W., and G.K. contributed to supervision and manuscript review. All authors reviewed and approved the final version of the manuscript.

## Data Availability

All data generated or analyzed during this study are included in this article and its online supplementary material. Further inquiries can be directed to the corresponding author.
